# Clinical outcomes after palbociclib with or without endocrine therapy in postmenopausal women with hormone receptor positive and HER2-negative metastatic breast cancer enrolled in the TREnd trial

**DOI:** 10.1186/s13058-019-1149-5

**Published:** 2019-05-29

**Authors:** Lorenzo Rossi, Chiara Biagioni, Amelia McCartney, Ilenia Migliaccio, Giuseppe Curigliano, Giuseppina Sanna, Erica Moretti, Alessandro M. Minisini, Saverio Cinieri, Carlo Tondini, Grazia Arpino, Antonio Bernardo, Angelo Martignetti, Emanuela Risi, Marta Pestrin, Luca Boni, Matteo Benelli, Laura Biganzoli, Angelo Di Leo, Luca Malorni

**Affiliations:** 1 “Sandro Pitigliani” Medical Oncology Department, Hospital of Prato, Prato, Italy; 2 Institute of Oncology of Southern Switzerland (IOSI), Bellinzona, Switzerland; 3 Breast Unit of Southern Switzerland (CSSI), Lugano, Switzerland; 4 Bioinformatics Unit, Hospital of Prato, Prato, Italy; 5 “Sandro Pitigliani” Translational Research Unit, Hospital of Prato, Prato, Italy; 6 Division of Early Drug Development, Department of Haematology and Haemato-Oncology, Istituto Europeo di Oncologia, IRCCS, Milano and University of Milano, Milan, Italy; 7 Department of Oncology, Azienda Sanitaria Universitaria Integrata di Udine, Udine, Italy; 8 Medical Oncology Department, ASL Brindisi, Brindisi, Italy; 9 Hospital Papa Giovanni XXIII, Bergamo, Italy; 10 Department of Clinical Medicine and Surgery, University of Naples Federico II, Naples, Italy; 11 Medical Oncology Department, ICS Maugeri IRCCS, Pavia, Italy; 12 Oncology Department, Azienda USL Toscana Sud Est, Hospital Alta Val D’Elsa, Poggibonsi, Siena Italy; 13 Clinical Trial Coordinating Center, AOU Careggi, Istituto Toscano Tumori, Florence, Italy

**Keywords:** Metastatic breast cancer, CDK4/6 inhibitors, Palbociclib, Best sequence, TREnd trial

## Abstract

Currently, there is limited data regarding the effectiveness of standard subsequent line therapies such as endocrine therapy, chemotherapy, or targeted agents after progression on CDK4/6 inhibitor-based regimens. This paper describes time-to-treatment failure beyond progression on palbociclib or palbociclib+endocrine therapy in patients enrolled in the phase II, multicenter TREnd trial. Our results indicate that there is limited benefit from post-palbociclib treatment, regardless of the type of therapy received. A small population of long responders were identified who demonstrated ongoing benefit from a subsequent line of endocrine therapy after progression to palbociclib-based regimens. A translational research program is ongoing on this population of outliers.

## Background

Recent developments in the management of metastatic breast cancer (mBC) have seen cyclin-dependent kinase 4 and 6 (CDK4/6) inhibitors, given in combination with endocrine therapy (ET), approved in the USA and in Europe for the first-line treatment of metastatic hormone receptor-positive (HR+)/human epidermal growth factor receptor-2 (HER2)-negative disease. Palbociclib (P), a selective inhibitor of CDK4/6, has shown significant activity in the first- or subsequent line treatment of HR+/HER2-negative mBC when combined with ET, resulting in an approximate doubling of progression-free survival (PFS) when compared to ET alone [1–3].

The TREnd trial is a multicenter phase II study that randomized 115 postmenopausal women with moderately pre-treated HR+/HER2-negative mBC (up to two lines of prior ET, and/or one line of chemotherapy (CT) for metastatic disease) to receive P alone, or P in combination with the ET received prior to progression [4]. The rationale of continuing the endocrine agent upon which disease had previously progressed was based on preclinical data suggesting palbociclib has the ability to reverse endocrine resistance [5]. The primary endpoint of TREnd demonstrated that P has significant clinical activity as a single agent. Exploratory analyses suggested that the combination of P with ET was better than P monotherapy in terms of duration of clinical benefit (11.5 months versus 6 months respectively; HR = 0.35; 95% CI 0.18–0.70, exploratory *p* value = 0.0021). A similar trend was also observed in terms of PFS. Further exploratory subgroup analyses demonstrated that PFS advantage was observed only in those patients with a previous durable response to ET (HR, 0.53; 95% CI, 0.3–0.9, exploratory *p* value = 0.02), providing the first clinical evidence that P may have potential to reverse acquired resistance to ET.

There is limited data regarding the effectiveness of standard subsequent line therapies (CT, targeted agents or ET) after progression on CDK4/6 inhibitor-based regimens, and all analyses thus far have been retrospective and exploratory in nature. Recent data have emerged from PALOMA-1 [6] and PALOMA-3 [7], suggesting that progression on P has no significant effect on the therapeutic benefit derived from subsequent treatments received off-trial. In this context, we conducted an analysis of prospectively collected data from patients enrolled in TREnd, in order to evaluate the efficacy of the subsequent line of therapy received after progression on the assigned trial arm, and the pattern of progression of disease.

## Patients and methods

The study design and baseline characteristics of enrolled patients on TREnd are described in detail elsewhere [4]. Briefly, at trial entry, three quarters of the overall population had pre-existing visceral disease, 69% had received only one prior line of ET for advanced disease, and 30% had also received one prior line of palliative CT. Most patients (73%) had received their most recent pre-trial line of endocrine therapy for more than 6 months.

The primary endpoint of the current analysis is the time-to-treatment failure (TTF) of the subsequent line of therapy received after TREnd, defined as the time interval between the commencement and discontinuation of next-line therapy for any reason. Additionally, we identified “long-responder” patients who had a duration of post-TREnd therapy (ET or CT) falling within the upper quartile. Clinical benefit (CB) was defined by the presence of a radiological complete response, partial response, or stable disease for at least 24 weeks according to RECIST 1.1 criteria. Overall survival (OS) was defined as the time from commencement of next-line therapy to death from any cause.

The cut-off date for the calculation of the TTF was April 24, 2018. TTF was summarized using the Kaplan-Meier method. Descriptive statistics were used. Statistical analyses were performed using R-software.

## Results

**Fig. 1:**
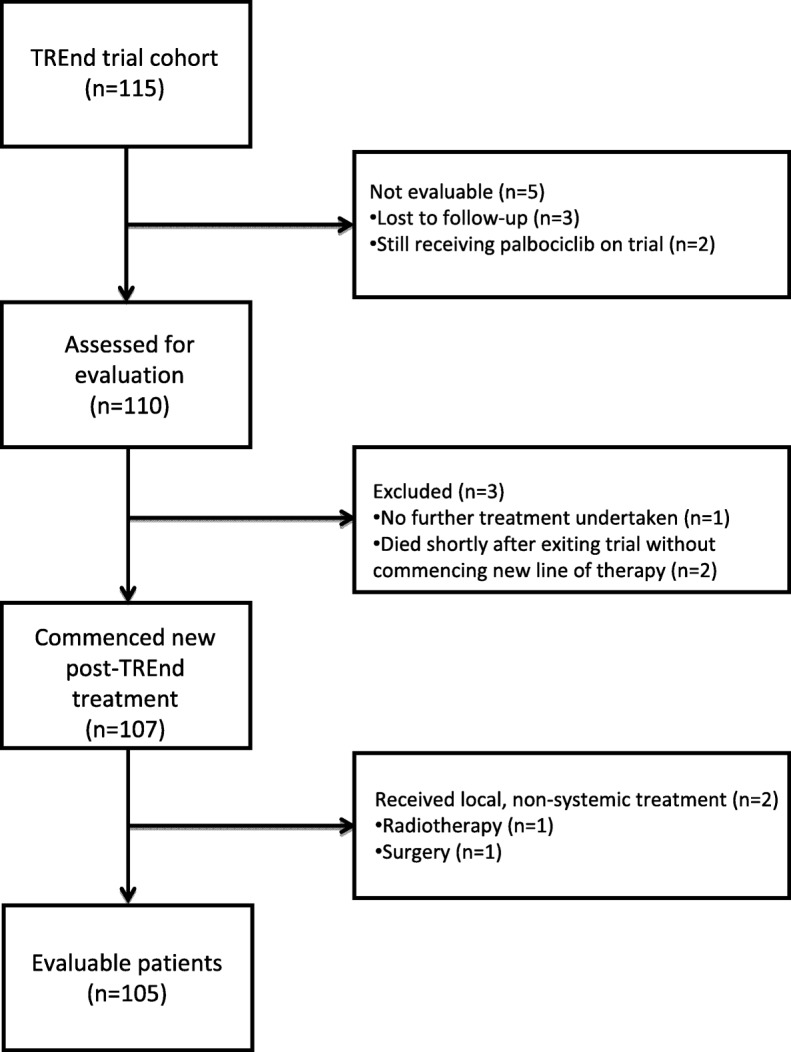
CONSORT diagram of evaluated patients

Of 115 patients enrolled into TREnd, we analyzed 105 patients with available follow-up information collected between October 2012 and September 2017, with a median follow-up of 25.8 months, estimated from the first day of commencing post-TREnd treatment. The median overall survival (mOS), with 52 events recorded, was 23.9 m (95% CI 18.9–33.8). Figure 1 reports the CONSORT diagram of evaluable patients.

After disease progression on TREnd, 9% of patients had bone-only disease, 76% had visceral involvement, and 15% had non-visceral disease (nodal +/− bone +/− skin metastases). Sixty-nine (66%) patients received CT as next-line treatment, 33 (31%) received ET, and 3 (3%) received novel or targeted therapies (TT) (Table 1).

**Table 1:** Type of systemic therapies employed as the immediate subsequent therapeutic line after TREnd (physician choice), classified by pharmacological classes

		Number
Subsequent chemotherapy (*n* = 69, 66%)		
Capecitabine-containing (*n* = 30)	Capecitabine	22
	Capecitabine + vinorelbine	6
	Capecitabine + vinorelbine + cyclophosphamide	2
Taxane-based (*n* = 21)	Paclitaxel	20
	Paclitaxel + BYL79*	1
Anthracycline-based (*n* = 9)	Doxorubicin	1
	Epirubicin	1
	Doxorubicin + cyclophosphamide	7
Platinum-containing (*n* = 2)	Cisplatin	1
	Cisplatin + cyclophosphamide	1
Others (*n* = 7)	Vinorelbine	3
	Cyclophosphamide + methotrexate	2
	Cyclophosphamide + vinorelbine	2
Subsequent endocrine therapy (*n* = 33, 31%)		
Fulvestrant (*n* = 20)		20
AIs (*n* = 7)	Letrozole	2
	Anastrozole	1
	Exemestane	4
AI + mTORi (*n* = 6)	Exemestane + everolimus	5
	Exemestane + everolimus + BYL719*	1
Subsequent other targeted/novel therapies (*n* = 3, 3%)		
Lucitanib		1
Ribociclib (single agent)		1
64-Cu-asparagine		1

*AIs* aromatase inhibitors, *mTORi* inhibitors of mammalian target of rapamycin

*BYL719 = alpelisib (PI3K inhibitor)

**Fig. 2:**
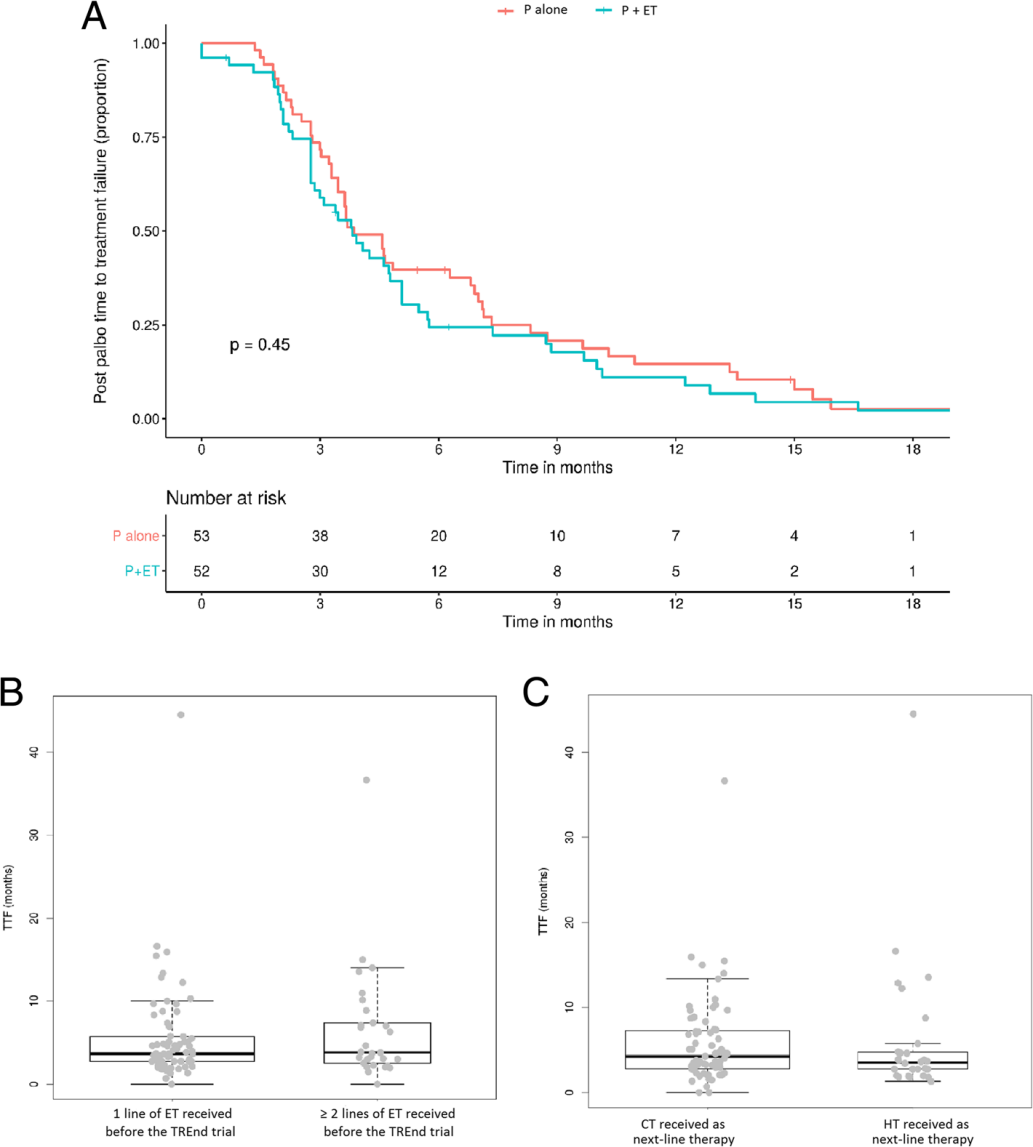
Kaplan-Meier curves of time to treatment failure (TTF) in the overall population (**a**). Box plots and comparison of TTF of patients who received only 1 or ≥ 2 lines of endocrine therapy prior to trial enrolment (**b**). Box plots and comparison of TTF of patients who received chemotherapy or endocrine therapy as next-line treatment (**c**). Abbreviations: P = palbociclib. ET = endocrine therapy. TTF = time to treatment failure. CT = chemotherapy

The overall median TTF of the next-line therapy was 3.8 months (m) (95% CI 3.5–4.8) and was unaffected by the arm to which the patient was randomized (Fig. 2a) (P single agent: mTTF 3.9 m, 95% CI 3.5–6.9 versus P + ET arm: 3.8 m, 95% CI 2.9–5.1; *p* = 0.45). Similarly, no significant differences in TTF were observed according to whether clinical benefit was attained on TREnd (3.7 m, 95% CI 3.3–5.1 for patients with CB, versus 4.01 m, 95% CI 3.2–7.1, for patients without CB; *p* = 0.26). Efficacy of post-TREnd therapy was also similar in patients, regardless of the number of ET lines received prior to trial enrolment (patients treated with one prior line, TTF 3.8 m, 95% CI 3.4–4.8 versus 3.9 m, 95% CI 3.1–7.4 in patients pre-treated with two prior lines, *p* = 0.57) (Fig. 2b). The mTTF according to absolute number of previous treatment lines received is reported in Table 2, with comparable findings. There was no significant difference in mTTF according to whether patients had received chemotherapy prior to enrolling in TREnd (3.82 m, 95% CI 3.45–5.72 in patients who were chemotherapy-naïve versus 3.85 m, 95% CI 2.86–5.07 in those who received prior chemotherapy; *p* = 0.15). Similar mTTF were observed in patients who received ET or CT post-TREnd (Fig. 2c) (patients who received ET: mTTF 3.7 months, 95% CI 2.8–4.8 versus patients who received CT: 4.6 months, 95% CI 3.5–5.8; *p* = 0.98).

**Table 2:** Median time to treatment failure on treatment regimen received immediately after TREnd, according to the absolute number of previous lines of treatment received. This takes into account previous endocrine therapy line(s) +/− chemotherapy (TREnd allowed subjects to have a maximum of one previous line of chemotherapy for advanced disease prior to enrolment)

Number of previous lines of treatment received	Patients (number)	Recorded events (number)	Median TTF (months)	95% CI
Two lines	51	47	3.73	3.39–5.07
Three lines	44	43	4.18	3.09–6.28
Four lines	10	9	3.85	2.20–NA

*TTF* time to treatment failure, *CI* confidence interval, *NA* not assessable

The mTTF of the next-line ET in patients from the P monotherapy arm was 4.6 m (95% CI 3.7–NA) versus 2.8 m in those previously enrolled in the combination arm (95% CI 2.8–5.7) (*p* = 0.36). Similarly, the mTTF of the next-line CT in patients from the P single-agent arm was 3.7 m (95% CI 3.3–7) versus 4.8 m (95% CI 3.8–7.4) in those from the P + ET arm (*p* = 0.57).

**Fig. 3:**
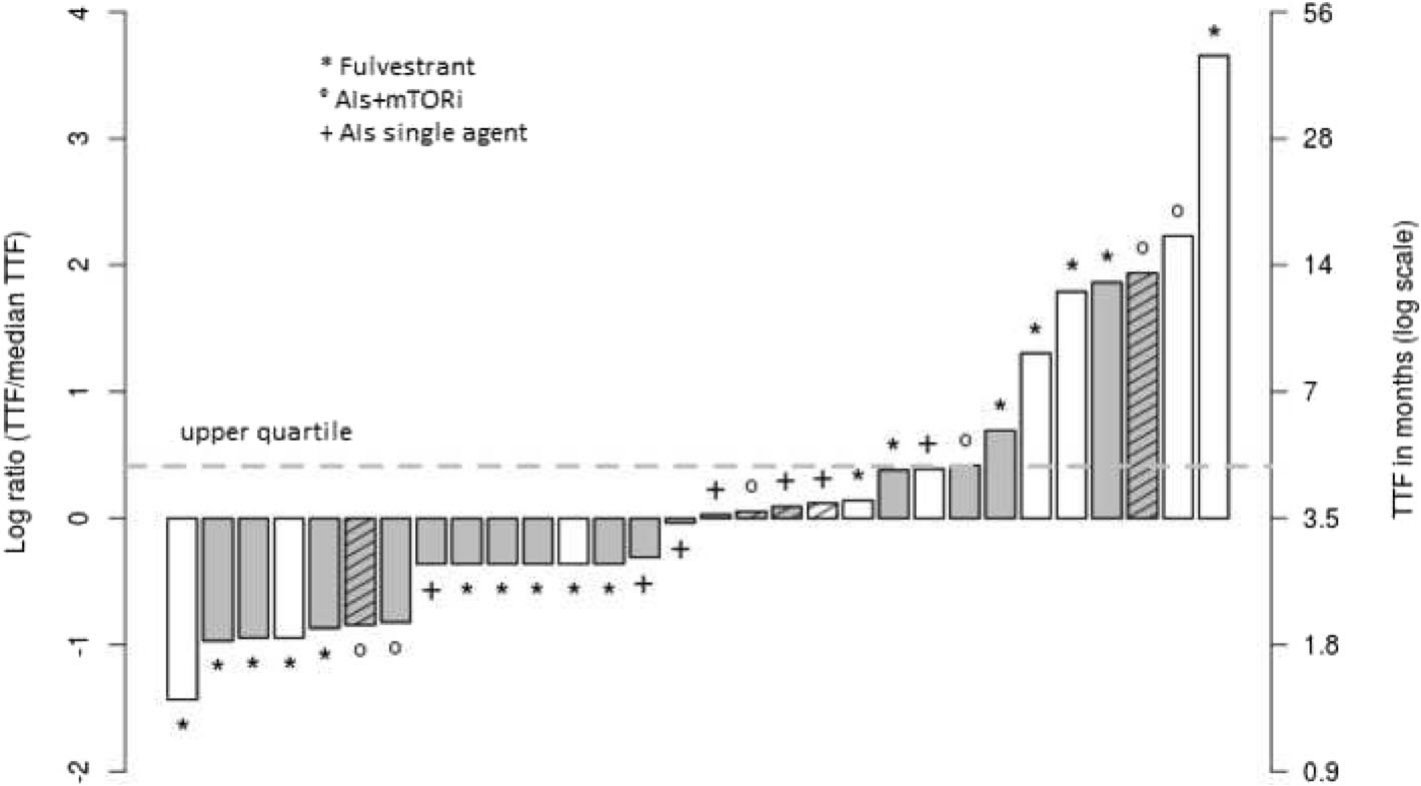
Bar plot of post-TREnd TTF in patients who received subsequent ET, scaled on a median value. Abbreviations: TTF = time to treatment failure. ET = endocrine therapy. AIs = aromatase inhibitors. mTORi = mTOR inhibitors. Values in *y*-axis are the logarithm of the relationship between single patient and median duration; 0 represents the median value of TTF. Dotted line: value of 3rd quartile (4.7 months). Gray columns denote patients who achieved clinical benefit shown on trial; white column: no clinical benefit demonstrated on trial. Hatched-lines column: denotes > 1 previous line of ET received before entering into the TREnd study

Twenty-five long-responding patients falling within the upper quartile of TTF on next-line therapy were identified (cut-off 4.7 m in patients who completed ET post-TREnd [range 4.7–44], and 7.2 m in those who completed chemotherapy [range 7.2–36]). Of these, 8 (32%) received ET, and 17 (68%) received CT immediately following TREnd. The mTTF of these long responders was 13 m (95% CI 12.2 9-NA) in those who received ET and 10 m (95% CI 8.9 10–15) in those who received CT. Figure 3 reports the characteristics of patients who received ET immediately following TREnd and identifies the long-responding patients.

## Discussion

CDK4/6 inhibitors have radically changed the management of HR+/HER2-negative mBC, with a significant improvement in median PFS when compared to ET alone [3, 8–12]. However, questions remain as to whether CDK4/6 inhibition can improve long-term OS, and which treatment approach is best to recommend following progression on CDK4/6 inhibitors. A comparative scarcity of data remains regarding both the effect P exerts on subsequent therapies such as CT and ET, and the clinical characteristics of progressive disease after successive lines.

In this retrospective evaluation of the dataset of TREnd, the median duration of the treatment received immediately post-palbociclib did not reach 5 months, and there were no substantial differences in TTF according to the post-palbociclib treatment received. These findings are similar to those reported in the PALOMA 1 and 3 cohorts [6, 7], suggesting that TTF following palbociclib may be independent of the magnitude of drug exposure prior to palbociclib. The overall next-line mTTF of 3.8 m observed in the TREnd dataset is in line with historical survival data in similar pre-treated study populations. In women enrolled in the EFECT trial, which enrolled patients with HR+ mBC progressing on a non-steroidal aromatase inhibitor to receive either exemestane or fulvestrant, the median time to progression was 3.7 m [13]. Fifty-eight percent of the women in EFECT had received at least two prior lines of ET, 23% had received palliative chemotherapy, and 57% had visceral disease involvement. Similarly, another study demonstrated the time to disease progression in women receiving capecitabine for previously anthracycline-treated, taxane-refractory mBC was 93 days [14]. In this population, 68% had a predominantly visceral burden of disease and on average had received at least two prior palliative chemotherapeutic regimens and one line of ET prior to trial entry. The mOS noted in the post-TREnd group is also not dissimilar to that observed in the PALOMA-3 population [7].

In this study, factors including the study arm to which the patient was allocated, clinical benefit observed on trial, and the agent used as next-line therapy (CT or ET) following progression on trial had little bearing on the duration of benefit derived from the next-line therapy. However, we observed some durable responses to post-TREnd therapy, albeit in a small number of patients. To identify and describe the clinical characteristics of these patients, we defined as “long responders” those who had a TTF in the upper quartile. Interesting data emerge from the eight long responders who received ET as next-line therapy. Of these, 7/8 received only one single line of ET before enrolment into TREnd, and 7/8 had received ET for > 6 months prior to randomization. These analyses, although exploratory and conducted on a small number of long responders, generate the hypothesis that a subgroup of patients that obtain CB from a previous ET may not have exhausted the full potential of ET, even after exposure to P. Therefore, continuing ET after P could lead to favorable outcomes in terms of response duration in certain subgroups of patients. The confounding factor remains our current inability to prospectively identify such groups. Furthermore, a deeper understanding of the molecular mechanisms of resistance may reveal information to identify the subgroups that may still derive benefit from continuing ET post-P. Somatic mutations of *ESR1* have been widely implicated in resistance to ET acquired under drug pressure and are conversely rarely ever identified in primary, ET-naïve BC [15]. Translational studies of samples collected in TREnd are ongoing and will include ctDNA analysis to study the incidence of somatic mutations in a panel of cancer genes including *ESR1* and their correlation with response to ET administered post study. Patients who demonstrated ongoing endocrine sensitivity after exiting TREnd may represent an *ESR1* wild-type population. Additional translational studies in the TREnd cohort include investigation into circulating markers of prognosis and early response to treatment and transcriptomic analyses of tumor samples.

In conclusion, our data suggest that subsequent therapies in patients with endocrine resistance who progress on palbociclib do not generally lead to durable responses, with the exception of some long responders who may represent a subgroup with partially preserved endocrine sensitivity. This highlights the need to develop new strategies to personalize management within patients with metastatic luminal disease. Additionally, our data supports the increasing need to integrate preclinical, translational, and clinical data in order to identify predictive markers and to develop treatment algorithms in HR+ HER2-negative mBC.

## Abbreviations

BC: Breast cancer
CDK4/6: Cyclin-dependent kinase 4 and 6
CI: Confidence interval
CONSORT: Consolidated Standards Of Reporting Trials
CT: Chemotherapy
ET: Endocrine therapy
HER2: Human epidermal growth factor receptor-2
HR: Hazard ratio
HR+: Hormone receptor positive
m: Months
mBC: Metastatic breast cancer
P: Palbociclib
PFS: Progression-free survival
TT: Targeted/novel therapies
TTF: Time-to-treatment failure
